# Active Principle Therapy

**Published:** 1910-03

**Authors:** Geo. L. Servoss

**Affiliations:** Fairview, Nevada


### Active Principle Therapy.

FAIRVIEW, NEVADA, October 20, 1909.

*Dr. F. E. Daniel, Editor Texas Medical Journal, Austin, Texas.*

MY DEAR DOCTOR: Your postal of the 14th, accepting my article on "Sanguinarine," was received yesterday and I am pleased to note that the article has been accepted and will appear at an early date in your journal. I do not know what your ideas may be on the subject of active principles, but after employing them to the exclusion of all other drug preparations for several years, I consider them far more satisfactory than the galenicals. I get better, quicker and surer results than I ever obtained with galenicals. When I began the use of active principles, like all others new in this system of therapy, I was, to a certain extent, fearful of my results, owing to the fact that it had been drummed into me that the active principles were too potent for employment. I had to get in and dig out what information I could obtain on the subject and found it hard work and it is owing to this fact that I am trying in a weak way to help in the education of the profession, particularly the younger men, as you say, to recognize the excellent features of this system. While much of the writing I do is from research work, I endeavor to always put my findings in my articles, and without throwing any bouquets at myself, I believe that these articles are doing the profession some good, as hardly one of them but has brought me one or more inquiries *in re* the subject upon which I have written. I sincerely believe that active principle therapy is to be the popular system within the very near future, as both the doctor and layman is recognizing the fact, more and more, that the galenicals are liable to be adulterated, and if not, are liable to decomposition. The pure food and drug laws are doing much to educate the people. I find that my patients take kindlier to the active principles in their pleasant form of administration than to the galenicals and that since I have learned not to be fearful of the action of them, I am getting results never obtained while using the older forms of the drugs. I am an enthusiast on the subject and like to bring my findings before the profession.

I congratulate you on the stand you have taken *in re* the "clique" of the A. M. A. I am a member of the Association, but I do not like the actions of Simmons, McCormack & Co. any more than you and the rest of the independents do, and I hope that we will soon see a reformation. The Association smacks too much of the personally owned sort of a thing to be popular with the average American. In 1776 we fought against taxation without representation and that fight is liable to be repeated in the A. M. A. I like the way Lydston comes out openly and defies the "peerless one." Lydston's work is not wholly in vain and I do not think it will be long until others will come forward with their assistance. I have gotten into the fight to a certain extent and would go into it further, but for very good reasons at this time. I can do better work in other ways than by openly attacking Simmons and his gang.

Thanking you for past favors, I am, with kindest regards,

Yours very sincerely,

GEO. L. SERVOSS, M. D.

I am enclosing a little article on "Lobelin" which comes in the same classification as do emetine and sanguinarine. I think this will be of interest to your readers. [Published herewith.]

